# Primate Assemblage Structure in Tanjung Datu National Park, Malaysian Borneo

**DOI:** 10.3897/BDJ.14.e159957

**Published:** 2026-05-15

**Authors:** Abd Rahman Mohd-Ridwan, Mohammad Noor-Faezah, Tukiman Nur-Aizatul, Roberta Chaya Tawie Tingga, Remond Tan, Azroie Denel, Badrul Munir Md-Zain

**Affiliations:** 1 Centre for Pre-University Studies, Universiti Malaysia Sarawak, Kota Samarahan, Malaysia Centre for Pre-University Studies, Universiti Malaysia Sarawak Kota Samarahan Malaysia; 2 Animal Resource Science and Management, Faculty of Resource Science and Technology, Universiti Malaysia Sarawak, Kota Samarahan, Malaysia Animal Resource Science and Management, Faculty of Resource Science and Technology, Universiti Malaysia Sarawak Kota Samarahan Malaysia; 3 Department of Biological Sciences and Biotechnology, Faculty of Science and Technology, Universiti Kebangsaan Malaysia, Bangi, Malaysia Department of Biological Sciences and Biotechnology, Faculty of Science and Technology, Universiti Kebangsaan Malaysia Bangi Malaysia; 4 Sarawak Forestry Corporation, Kota Sentosa, Kuching, Malaysia Sarawak Forestry Corporation, Kota Sentosa Kuching Malaysia

**Keywords:** primate, diversity, distribution, conservation, Borneo

## Abstract

Understanding primate assemblage structure in small protected areas is important for effective conservation planning, particularly in biodiversity-rich regions such as Malaysian Borneo. This study assessed primate species composition, encounter rates, distribution and site occupancy in Tanjung Datu National Park (TDNP), Sarawak. Systematic diurnal and nocturnal surveys were conducted from July 2023 to February 2024 along four main trails, covering 420 survey hours and approximately 8.9 km of transects. A total of 226 individuals, representing eight species from four families were recorded. *Macaca fascicularis* showed the highest encounter rate, followed by *Trachypithecus cristatus*. The Critically Endangered Bornean banded langur (*Presbytis chrysomelas chrysomelas*) was detected across all survey site, but occurred at relatively low encounter frequencies. Species richness and diversity indices indicated moderate variation amongst sites, although the differences were not statistically significant. Riverine and coastal forest habitats supported the highest primate diversity. The occurrence of several threatened species highlights the conservation importance of TDNP within an increasingly fragmented coastal landscape. These findings provide important baseline information for future primate monitoring and support improved conservation management and sustainable nature-based tourism planning in the Park.

## Introduction

Malaysia is recognised as a global biodiversity hotspot and supports one of the richest primate assemblages in Southeast Asia (Holzner et al. 2021). Its extensive tropical forest ecosystems provide suitable habitats for primates belonging to five families: Lorisidae, Tarsiidae, Cercopithecidae, Hylobatidae and Hominidae. Early regional assessments highlighted the high diversity and ecological importance of primates within Southeast Asian tropical forests (Mittermeier 1988). Subsequent advances in taxonomy and molecular systematics have further improved the understanding of primate diversity in the region (Brandon-Jones et al. 2004; Md-Zain et al. 2022). Current estimates indicate that Malaysia supports approximately 26 primate species representing nine genera (Md-Zain et al. 2022). Of these, Malaysian Borneo harbours 15 species from eight genera, while Peninsular Malaysia supports 13 species from six genera (Md-Zain et al. 2022). Many of these species occur within protected areas that function as important refuges for biodiversity and are increasingly used for nature-based tourism.

Primates play important ecological roles in tropical forest ecosystems, particularly in seed dispersal, herbivory and maintaining forest regeneration processes (Chapman and Dunham 2018). Despite this ecological importance, primate populations across Malaysia are increasingly threatened by anthropogenic pressures. Habitat loss, forest degradation and fragmentation remain the primary drivers of primate population decline, often accompanied by hunting, illegal wildlife trade, road mortality and human primate conflict (Lappan and Ruppert 2019; Matsuda et al. 2020;Boonratana et al. 2022). National assessments indicate that many primate species in Malaysia are currently threatened or experiencing population declines (Nur-Aizatul et al. 2023). These trends highlight the need for updated field-based information on primate diversity, distribution and population status, particularly within protected areas that serve as conservation strongholds.

Sarawak supports a high diversity of primates, with 14 species recorded across various forest ecosystems in the state (Md-Zain et al. 2022). Several protected areas in Sarawak have been recognised as important sites for primate research and conservation. For example, Bako National Park is well known for studies on the proboscis monkey, while Samunsam Wildlife Sanctuary and Maludam National Park have also been identified as important habitats for several threatened primates, including Bornean banded langur and proboscis monkey (Nur-Aizatul et al. 2023). Although these areas have received increasing research attention, primate diversity assessments remain uneven across protected areas in the region and several parks still lack detailed baseline ecological information.

Tanjung Datu National Park (TDNP), located in south-western Sarawak near the border with West Kalimantan, Indonesia, represents an important coastal forest ecosystem in the region (Hazebroek and Abang Morshidi 2006). The Park encompasses a mosaic of habitats, including lowland rainforest, hill forest and coastal vegetation, which support diverse wildlife communities. In recent years, TDNP has also emerged as a destination for nature-based tourism, with most visitor activities concentrated along designated forest trails and areas surrounding the Park headquarters (Abdul Rahman et al. 2015).

Several ecological and population studies on primates have been conducted in TDNP (Ampeng 2003, Ampeng 2007, Noor-Faezah et al. 2023, Nur-Aizatul et al. 2024, Nur-Aizatul et al. 2025, Noor-Faezah et al. 2025, Arifin et al. 2026), documenting the presence of multiple primate species within the Park. However, despite these research efforts, systematic information on primate diversity and encounter patterns remains limited. Most available records are derived from general biodiversity surveys or incidental observations and comprehensive data on species occurrence and encounter rates are still lacking. Such baseline ecological information is essential for evaluating the conservation importance of the Park and supporting evidence-based management.

Therefore, this study aimed to assess primate diversity in TDNP by documenting species composition, distribution within the Park and encounter rates along forest trails and areas surrounding the Park headquarters. The findings provide updated baseline information on the primate assemblage of TDNP and contribute to improved biodiversity monitoring, conservation planning and sustainable management of protected areas in coastal forest ecosystems of Sarawak. As one of the least studied coastal protected areas in the region, understanding primate assemblages in TDNP is essential for strengthening conservation strategies and safeguarding primate diversity in Malaysian Borneo.

## Material and methods

### Study site

**Figure 1.**
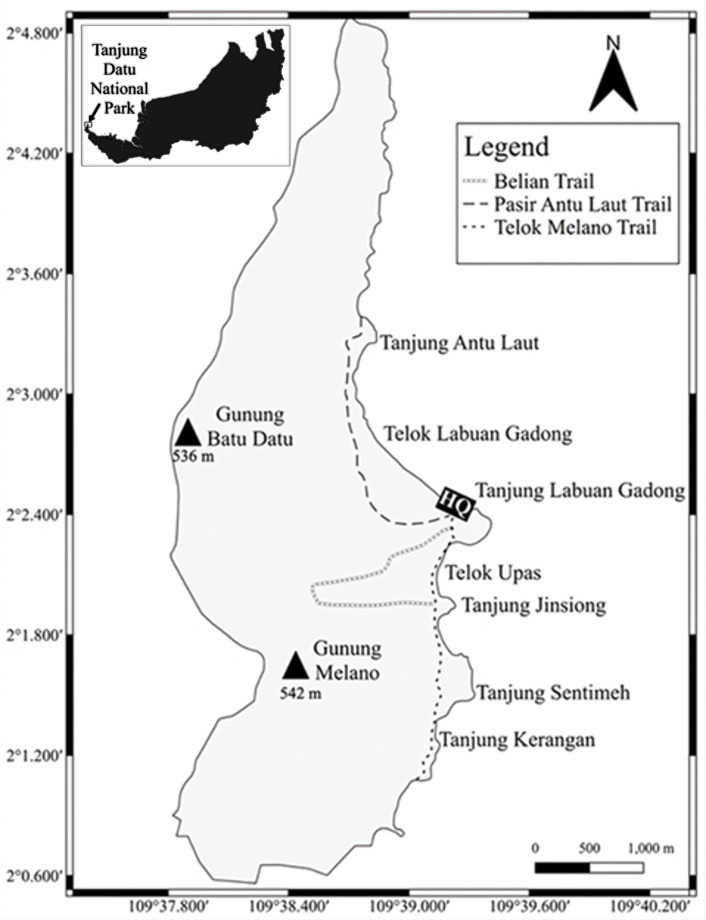
Map of TDNP with trails.

Tanjung Datu National Park (TDNP), is one of the smallest national parks in Sarawak. It is located at the westernmost tip of Sarawak, at 2°02′N and 109°38.5′E, with an area of 13.79 km^2^ (Mohd-Azlan et al. 2018). It lies on the Datu Peninsula, along the boundary of Malaysia and Indonesia. Gazetted in 1994, the Park has been accessible to tourists following post-pandemic COVID-19 and has been one of the most popular tourist destinations ever since (Lo et al. 2015). TDNP can be accessed through a 30-minute boat journey or a 4-hour jungle trek via the Telok Melano Trail, which is 3.7 km long (Lo et al. 2015). The Park is rich in biodiversity, albeit the area is small. White sandy beaches, crystal clear ocean and streams surround TDNP. Furthermore, the vegetation consists of dipterocarp forest, with some ridge-top forest along the mountain ridge over 400 m elevation. The primary survey locations covered four sites: Headquarters (HQ) Pasir Antu Laut Trail (PALT), Belian Trail (BT) and Telok Melano Trail (TMT) (Fig. 1).

### Field Sampling and Survey Design

Primate surveys used a direct encounter-based count along four trail transects: PALT (2.7 km), TMT (3.7 km), BT (2.0 km) and HQ (0.5 km), totalling 8.9 km. All individuals detected were recorded by species and site. For each encounter, species identity, detection time, location (GPS coordinates) and group size (when observable) were recorded. Surveys were carried out between July 2023 and February 2024, excluding October and December 2023 when access to TDNP was restricted due to the off-season. A total of 420 hours of direct observation were completed, with approximately 10 hours of survey effort per sampling day.

#### Diurnal Surveys

Diurnal surveys were conducted daily between 0630 h and 1830 h, encompassing early morning and afternoon periods when primate activity is typically highest. Observations focused on visual detection of canopy movement and auditory cues, particularly vocalisations. Special attention was given to early morning calling activity of Hylobatidae and late afternoon vocalisations of Cercopithecidae. Upon detection, species identity, group size, behaviour and GPS coordinates were recorded. Binoculars were used to aid visual detection and a digital single-lens reflex (DSLR) camera was used to capture photographs, videos and audio recordings for species verification. To enhance detection probability and confirm species presence, acoustic playback was conducted opportunistically following suspected detections. Pre-recorded calls of target species were broadcast up to five times per species, with a 30-s interval between playbacks to observe behavioural or vocal responses, following established protocols (Clink et al. 2017).

#### Nocturnal Surveys

Nocturnal surveys targeting nocturnal primate species were conducted daily between 2200 h and 0100 h along the same forest trails to ensure consistency in spatial coverage. Observers walked slowly and quietly while scanning the understorey and canopy using red-light headlamps and handheld torch lights to detect eye-shine and movement. The use of red light minimised disturbance while maintaining sufficient visibility for detection, consistent with standard nocturnal primate survey methods (MacKinnon and MacKinnon 1980, Bearder et al. 2006, Nekaris et al. 2008). Upon detection, species identity and location were recorded and visual confirmation was attempted where possible.

#### Equipment and Data Recording

Field equipment included a digital single-lens reflex (DSLR) camera for visual and audio documentation, a global positioning system (GPS) unit for precise location recording and binoculars to facilitate detection of arboreal species. All encounters were geo-referenced and survey type (diurnal or nocturnal) and detection time were recorded to support subsequent analyses of encounter patterns.

### Data analysis

Encounter rate (ER) was calculated as the number of individuals recorded per kilometre surveyed (individuals km⁻¹) (Johnson et al. 2023), while site occupancy was defined as the proportion of surveyed sites in which a species was detected. Primate species richness was assessed using the Shannon–Wiener diversity index (*H*’). As the Shapiro–Wilk test indicated non-normal data distribution (p < 0.05), differences in primate diversity amongst habitat types were tested using the non-parametric Kruskal–Wallis H test (Bernard et al. 2016). All statistical analyses were conducted using PAST oftware (Hammer et al. 2001). A heatmap illustrating primate distribution across sites in TDNP was generated using ClustVis software (Metsalu and Vilo 2015).

## Results

**Figure 2.**
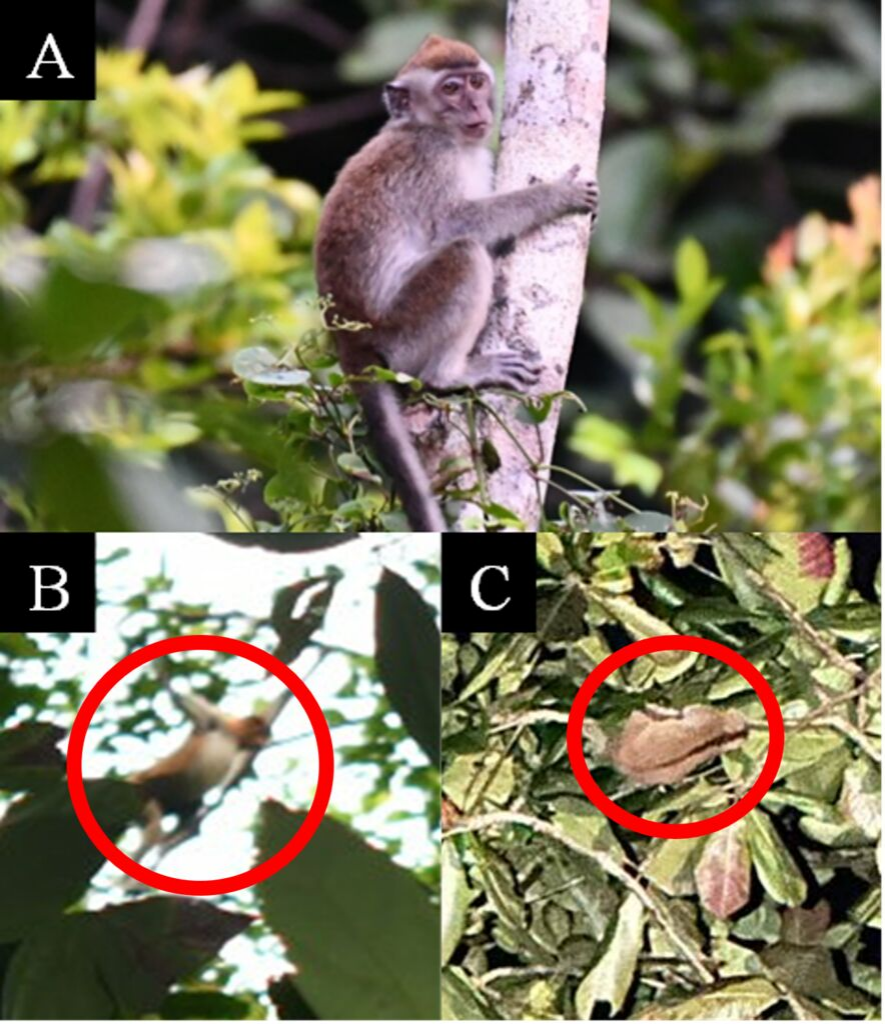
Primate species encountered in TDNP. **A** Long-tailed macaque (*Macaca fascicularis*); **B** Proboscis monkey (*Nasalis larvatus*); **C** Philippine slow loris (*Nycticebus menagensis*) highlighted in the red circle.

**Figure 3.**
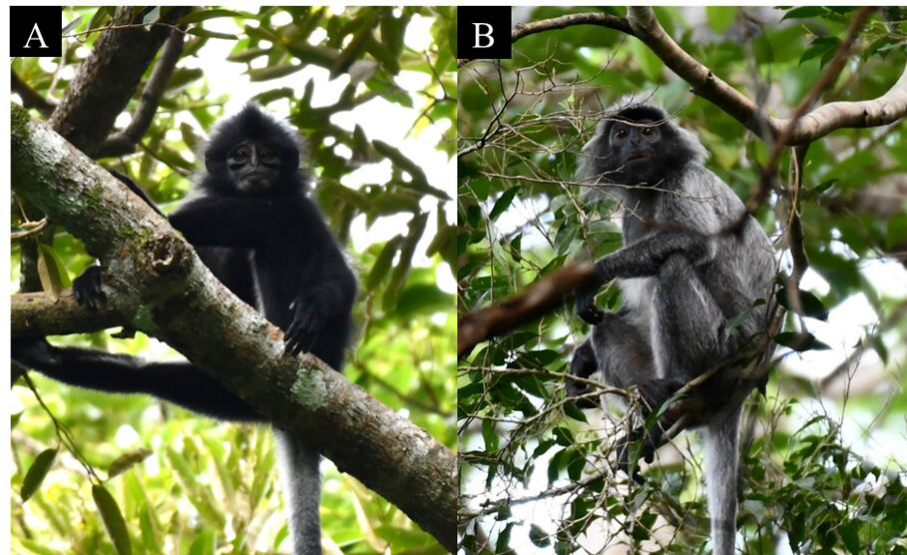
Detected primate species in TDNP via direct observation and vocalisation. **A** Bornean banded langur (*Presbytis chrysomelas chrysomelas*); **B** Silvery langur (*Trachypithecus cristatus*).

A total of 226 individuals of primates were recorded at TDNP, encompassing eight primate species from four distinct families, namely Lorisidae, Tarsiidae, Hylobatidae and Cercopithecidae. Five primate species were observed directly, namely *Macaca fascicularis*, *Macaca nemestrina*, *Nasalis larvatus*, *Nycticebus menagensis* and *Cephalopachus bancanus* (Fig. 2), while the other three species were observed directly and detected indirectly based on vocalisation: *Hylobates abbotti*, *Presbytis chrysomelas chrysomelas* and *Trachypithecus cristatus* (Fig. 3).

The species most frequently encountered within TDNP is *M. fascicularis* (n = 101; ER: 11.35 km-¹), followed by *T. cristatus* (n = 63; 7.08 km-¹), *P. c. chrysomelas* (n = 21; 2.36 km-¹), *H. abbotti* (n = 17; 1.91 km-¹), *M. nemestrina* (n = 11; 1.24 km-¹), *N. larvatus* (n = 5; 0.56 km-¹), *N. menagensis* (n = 5; 0.56 km-¹) and *C. bancanus* (n = 3; 0.34 km-¹) (Table 1). According to the IUCN Red List of Threatened Species, one species (*P. c. chrysomelas*) is Critically Endangered (Nijman et al. 2020) and five species (*C. bancanus*, *M. fascicularis*, *M. nemestrina*, *N. larvatus* and *H. abbotti*) are categorised as Endangered (Cheyne and Nijman 2020, Shekelle and Yustian 2020, Boonratana et al. 2021, Hansen et al. 2022, Ruppert et al. 2022). In addition, *N. menagensis* and *T. cristatus* are classified as Vulnerable (Meijaard and Nijman 2020, Nekaris et al. 2020).

**Figure 4.**
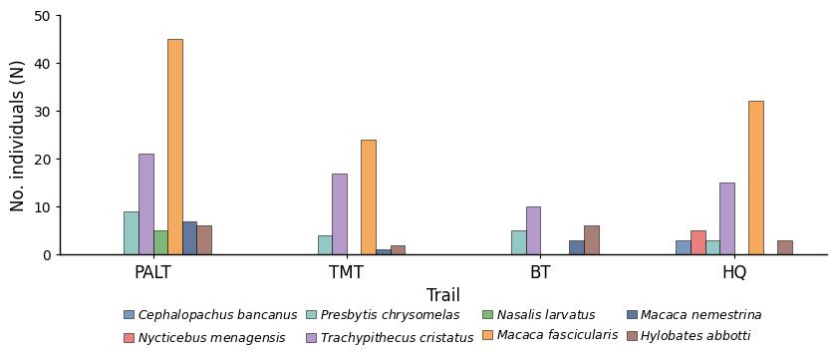
Species abundance and distribution of primates across four survey trails in Tanjung Datu National Park. Bars represent the number of individuals recorded per species at each trail. *Note*: Pasir Antu Laut Trail (PALT), Telok Melano Trail (TMT), Belian Trail (BT), Headquarters (HQ).

The findings highlight the significance of *P. c. chrysomelas*, which is the most frequent primate species detected across all four sites alongside *T. cristatus* and *H. abbotti*. Meanwhile, *M. fascicularis* showed the highest numbers of individuals observed within TDNP (Table 1; Fig. 4). Amongst the study areas, only the PALT and the HQ area served as habitats for six different primate species, representing a high level of primate diversity. The PALT was home to *P. c. chrysomelas*, *T. cristatus*, *M. fascicularis*, *H. abbotti*, *M. nemestrina* and *N. larvatus* (Fig. 4). Meanwhile, the HQ area recorded sightings of *C. bancanus*, *N. menagensis*, *P. c. chrysomelas*, *T. cristatus*, *M. fascicularis* and *H. abbotti* (Fig. 4). These species are commonly sighted within the riverine forest of the PALT and the beach forest near the HQ area. Five species of primates which include *T. cristatus*, *M. fascicularis*, *P. c. chrysomelas*, *H. abbotti* and *M. nemestrina*, were spotted at TMT along the beach forest. In contrast, BT had the fewest species, with only four species present, namely *H. abbotti*, *P. c. chrysomelas*, *T. cristatus* and *M. nemestrina*. Notably, the lowlands, below 200 metres above sea level, had the highest number of primate species, while the riverine forest and coastal forest habitats exhibited the most remarkable species diversity. The hilly areas of TDNP had significantly fewer primate species.

**Figure 5.**
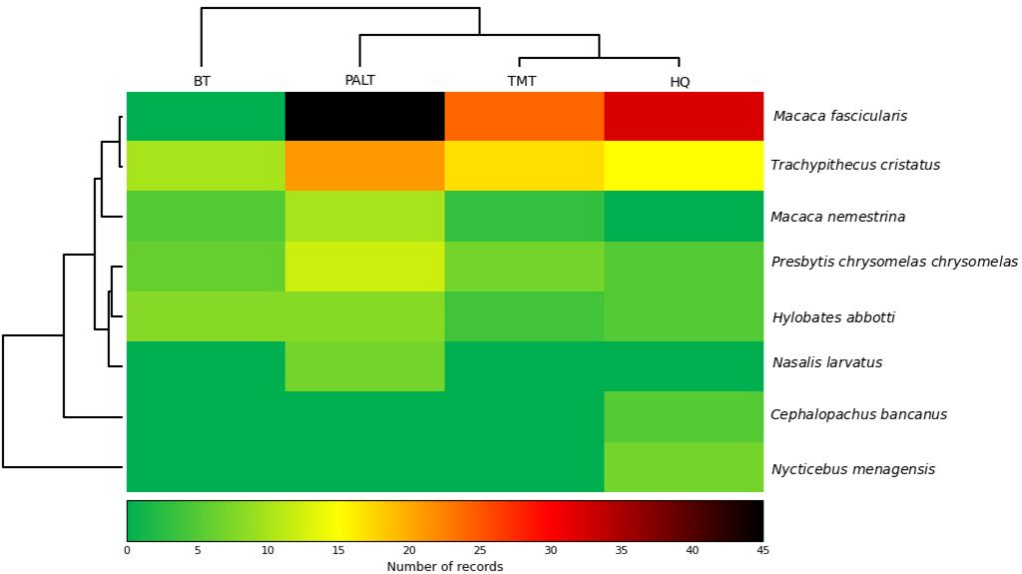
Heat map of primate records across survey sites in Tanjung Datu National Park. Rows and columns are hierarchically clustered, based on occurrence patterns. Colour intensity indicates the number of records per species at each site. Note: Pasir Antu Laut Trail (PALT), Telok Melano Trail (TMT), Belian Trail (BT), Headquarters (HQ).

The heatmap shows the number of primate encounter records across four survey sites. *Macaca fascicularis* shows the highest encounter numbers, particularly at HQ and TMT, while *T. cristatus* is also frequently recorded across all sites. Other diurnal primates, including *P. c. chrysomelas*, *H. abbotti* and *M. nemestrina*, display moderate and more even distributions. Nocturnal species (*C. bancanus* and *N. menagensis*) have consistently low values and are mainly recorded at HQ. Site clustering indicates that TMT and HQ have similar encounter patterns, whereas BT differs from the other sites (Fig. 5).

Amongst study sites, both PALT and HQ reported sightings of six primate species. Yet, PALT recorded the highest Shannon–Wiener diversity index value (*H*’ = 1.442), indicating greater primate diversity than the other survey sites. Conversely, TMT recorded the lowest highest *H*’ index (*H*’ 1.134). The Kruskal-Wallis test unveiled the primate diversity amongst the four areas are statistically equivalent as the p-value (0.455) > 0.05. Thus, the diversity of primate species in PALT, TMT, BT and HQ has no significant difference. Although BT recorded the lowest number of primate individuals (n = 24), it exhibited the highest species evenness (E = 0.916), followed by PALT (E = 0.705) and HQ (E = 0.632), indicating a relatively balanced primate assemblage at BT. In contrast, TMT recorded the lowest evenness value (E = 0.622) and a low Shannon–Wiener diversity index, reflecting lower diversity and the dominance of fewer species.

## Discussion

This study confirmed the presence of eight primate species in Tanjung Datu National Park (TDNP). This represents more than half of the primate species recorded in Sarawak (Nur-Aizatul et al. 2023) and indicates higher species richness than previously reported for the Park (Ampeng 2003). The greater number of species detected is likely due to broader survey coverage and repeated sampling across different habitat types and trail systems, highlighting the importance of systematic and well-planned surveys, particularly in small protected areas. Species detection was also supported by distinct vocalisations, which aided field identification. For example, *H. abbotti* produces unique duet calls that are easily recognised, *P. c. chrysomelas* can be identified by its characteristic “tat-tat-tat-tat” call (Ampeng et al. 2024) and *T. cristatus* emits an “ehok-ehok” call, typically produced by the adult male. Although TDNP supports relatively high primate diversity, encounter rates were uneven, with a few species accounting for most observations, while others were recorded less frequently.

Species with high ecological adaptability and tolerance to human disturbance, particularly *M. fascicularis* and *T. cristatus*, recorded the highest encounter rates and were detected across most study sites. Their frequent observations along trails and near the Park headquarters suggest that these species commonly use edge habitats and areas influenced by human activities. Similar patterns have been reported in Bukit Melawati, Kuala Selangor, where both species forage together near forest edges and obtain food from visitors, increasing their visibility during surveys (Mohd-Daut and Effendy 2022). This trend has also been observed in Bako National Park, where both species are amongst the most frequently sighted primates (Wan Azman and Khan 2022). These results suggest that encounter rates in TDNP reflect not only population size, but also behavioural characteristics, such as habituation to humans, dietary flexibility and the ability to exploit modified habitats.

In contrast, forest-dependent and habitat-specific species showed more restricted spatial distributions and lower encounter rates. *Nasalis larvatus* was recorded only at Pasir Antu Laut, indicating limited availability of suitable coastal–riverine and mangrove habitats within TDNP. In areas with larger and more continuous mangrove forests, higher population densities have been reported (Ampeng 2003). This pattern aligns with findings from other parts of Borneo, where *N. larvatus* showed the lowest detection rate and was found in a single site within the heavily logged forest (Bernard et al. 2016). Similarly, the nocturnal primates, *C. bancanus* and *N. menagensis*, were each detected at a single site, likely due to small population size and reduced detectability related to tall canopy structure, limited spotlight range and lower nocturnal survey effort.

A key finding of this study is the presence of the critically endangered *P. c. chrysomelas* at all surveyed sites. However, encounter frequencies were low compared with more generalist primate species, suggesting that the population density within the Park is relatively small. This pattern is consistent with the species’ ecological preference for tall-canopy, undisturbed forests and its low tolerance to anthropogenic disturbance (Noor-Faezah et al. 2023). The occurrence of this species across different habitat types in TDNP is also consistent with recent surveys conducted in the Park (Nur-Aizatul et al. 2024). Previous studies have further demonstrated that *P. c. chrysomelas* has a highly restricted distribution in Sarawak, highlighting the important role of TDNP in supporting the conservation of this threatened taxon.

The occurrence of multiple threatened primate species, including *P. c. chrysomelas* and *N. larvatus*, indicates that TDNP serves as a critical refuge within an increasingly fragmented coastal landscape. The findings highlight both opportunities and challenges for TDNP management. High encounter rates of adaptable generalist species in proximity to visitor hotspots enhance wildlife observation opportunities. Reliable primate sightings, like *N. larvatus* in Bako National Park (Wan Azman and Khan 2022), *Pongo pygmaeus* in Batang-Ai National Park (Pandong et al. 2018), *M. fascicularis* and *T. cristatus* across Sarawak ecotourism sites, demonstrate how ecotourism hotspots can offer exceptional wildlife experiences. However, this proximity also poses the risk, including increased human–primate interactions as seen in Sepilok Orangutan Rehabilitation Centre, where *M. fascicularis* displayed vigilance behaviour towards the tourist (Gilhooly et al. 2021). Behavioural shifts, such as food theft and chasing visitors, are reported amongst *M. fascicularis* at Batu Caves, indicating increasing tolerance and potential food-associated conditioning (Osman et al. 2022, Zamri and Md-Zain 2022).

Despite these pressures, TDNP retains high conservation value, as demonstrated by the persistent presence of threatened and forest-dependent species across multiple sites. Maintaining this value requires protection of forest integrity and habitat connectivity. Management strategies should balance visitor access with conservation goals. Key actions include regulating tourism intensity along sensitive trails, reducing habitat disturbance and discouraging wildlife feeding. Long-term monitoring that integrates encounter rates, occupancy analyses and complementary approaches, such as camera trapping or environmental DNA, will further strengthen evidence-based management and support sustainable tourism planning.

## Conclusions

This study provides an updated checklist of primates in TDNP, documenting eight species from four families, based on 420 hours of field surveys. The findings emphasise the Park’s high conservation value, particularly through the presence of several threatened primates, including the Critically Endangered *P. c. chrysomelas*. Despite its relatively small size and increasing tourism pressure, the Park continues to function as an important refuge for primates in Malaysian Borneo. However, the low encounter rates and limited distribution of several threatened species indicate that these populations remain vulnerable. Continued monitoring, habitat protection and the maintenance of forest connectivity are, therefore, essential to support long-term primate conservation in the Park.

## Figures and Tables

**Table 1.** Primate species composition, encounter rate (ER), occupancy and conservation status in Tanjung Datu National Park. Values represent the number of individuals recorded at each site. *Note*: Pasir Antu Laut Trail (PALT), Telok Melano Trail (TMT), Belian Trail (BT), Headquarters (HQ), International Union for Conservation of Nature (IUCN), Totally Protected (TP); Protected (P), Endangered (EN), Vulnerable (VU), Critically Endangered (CR).

**No.**	**Family Scientific Name**	**N**	**PALT**	**TMT**	**BT**	**HQ**	**ER (individuals km⁻¹)**	**Occupancy** **(%)**	**IUCN**
	** Tarsiidae **								
1	*Cephalopachus bancanus*	3	0	0	0	3	0.34	25	EN
	** Lorisidae **								
2	*Nycticebus menagensis*	5	0	0	0	5	0.56	25	VU
	** Cercopithecidae **								
3	*Presbytis chrysomelas chrysomelas*	21	9	4	5	3	2.36	100	CR
4	*Trachypithecus cristatus*	63	21	17	10	15	7.08	100	VU
5	*Nasalis larvatus*	5	5	0	0	0	0.56	25	EN
6	*Macaca fascicularis*	101	45	24	0	32	11.35	75	EN
7	*Macaca nemestrina*	11	7	1	3	0	1.24	75	EN
	** Hylobatidae **								
8	*Hylobates abbotti*	17	6	2	6	3	1.91	100	EN
	*H*’ index	1.51	1.442	1.134	1.298	1.333			
	Species evenness (E)	0.56	0.705	0.622	0.916	0.632			
	Total Individuals (N)	226	93	48	24	61			
	Total species	8	6	5	4	6			
